# On methodological approaches for non‐invasive characterization of muscle oxygen uptake recovery kinetics

**DOI:** 10.1113/EP094185

**Published:** 2026-11-06

**Authors:** Alessio Marciano, Harry B. Rossiter

**Affiliations:** ^1^ Respiratory Research Center The Lundquist Institute for Biomedical Innovation at Harbor‐UCLA Medical Center Torrance California USA; ^2^ Division of Respiratory and Critical Care Physiology and Medicine, Department of Medicine Harbor‐UCLA Medical Center Torrance California USA

**Keywords:** mitochondrial function, muscle, near‐infrared spectroscopy, oxygen kinetics

1

To the Editor,

We read with interest the expanding body of literature using near‐infrared spectroscopy (NIRS) to measure muscle oxygen uptake recovery kinetics. Two recent papers in *Experimental Physiology* using this technique warrant further discussion (Costalat et al., 2025; Pelka et al., 2026).

Pioneering studies by Hamaoka et al. (1996), Motobe et al. (2004) and Ryan et al. (2012) introduced and refined a NIRS‐based method to estimate muscle oxygen uptake ($m{\dot{V}}_{O_{2}}$) recovery kinetics. Using intermittent arterial occlusions, relative $m{\dot{V}}_{O_{2}}$ can be measured from the slope of tissue deoxygenation over time, and $m{\dot{V}}_{O_{2}}$ kinetics can be characterized by its recovery rate constant (*k*).

This approach is important because $m{\dot{V}}_{O_{2}}$ *k* is proportional to muscle oxidative capacity, allowing a simple, inexpensive and non‐invasive window on health‐related muscle mitochondrial function. NIRS‐based assessment of muscle oxidative capacity has strong construct validity and strong agreement with independent estimates of muscle oxidative capacity, such as citrate synthase activity, phosphocreatine recovery *k* (*r* = 0.88–0.95; Ryan et al., 2013) and maximal O_2_ flux in permeabilized fibre bundles (*r* = 0.61–0.74; Ryan et al., 2014). As use of this approach has grown, many variations of the original methods have been developed. Two recent studies in *Experimental Physiology* provide examples of developing methodological frameworks.

The protocol used by Pelka et al. (2026) included a delay of ∼15 s between the end of exercise and the first arterial occlusion and was followed by a time‐based occlusion sequence. This approach is suboptimal because it misses the most dynamic portion of $m{\dot{V}}_{O_{2}}$ recovery. Measurements made later in the transition carry limited kinetic information. Instead, optimal protocols would maximize the frequency of measurement in the early dynamics, where the kinetic information is richest. These technical limitations might negatively impact the validity of *k* as an estimate of oxidative capacity. Indeed, the reported baseline *k* (∼1.0 min^−1^) is approximately half of that expected for healthy individuals (∼2.0 min^−1^).

Costalat et al. (2025) used a 3 s segmental analysis within each 6 s occlusion with the aim to identify the most reliable method of *k* measurement. The conclusion was that mean differences among values of *k* were trivial (although reliability differed). This supports the concept that prolonged arterial occlusions are not necessary, allowing for brief (e.g., 3 s or less), high‐frequency measurements during the early $m{\dot{V}}_{O_{2}}$ recovery phase to optimize the kinetic information gained.

Additionally, individual deoxygenation slopes are strongly mediated by local oxygenation. For example, the deoxygenation slope measured below ∼50% of the individual's physiological range is shallower than that conducted in well‐oxygenated tissue (Pilotto et al., 2022). This raises several points of interest. Deviations of deoxygenation slope could reflect: (1) incomplete venous and/or arterial occlusion; (2) failure to correct for shifts in microvascular total heme; (3) limitation to mitochondrial O_2_ delivery; (4) technical or instrumental variability; and/or (5) non‐constant $m{\dot{V}}_{O_{2}}$.

The reductions in deoxygenation slope within a single occlusion observed by Costalat et al. (2025) are, therefore, expected, because $m{\dot{V}}_{O_{2}}$ is recovering in an approximately exponential manner over time. This effect would be greatest early in the recovery process and is expected to be more evident the longer duration of each arterial occlusion (assuming that O_2_ availability does not become limiting). Given the measured recovery kinetics (*k* ∼2.5 min^−1^), $m{\dot{V}}_{O_{2}}$ recovery in the study by Costalat et al. (2025) is expected to be ∼86% completed within the first three occlusion cycles (6 s occlusion, 10 s release). Thus, the relatively slow duty cycle of cuff occlusion and release is another example of a protocol that does not optimize the frequency of measurement during the dynamic phase of the $m{\dot{V}}_{O_{2}}$ recovery kinetics.

Together, these findings suggest that time‐based approaches to arterial occlusions during the NIRS‐based muscle oxidative measurement are suboptimal from both a measurement frequency perspective and because they have the potential to introduce saturation dependence of the physiology underlying *k*.

Instead, we advocate a protocol that aims both to increase the number of measurements in the dynamic phase and to control the tissue saturation using a variation of the saturation‐clamp approach proposed by Pilotto et al. (2022) and Villanova et al. (2025). Here, moderate‐intensity exercise is used initially to control the tissue saturation close to the target. This can be achieved with small variations in exercise duration or exercise duty cycle, meaning that occlusions can begin without a delay after the end of exercise. Then repeated occlusions are performed with variable durations of occlusion and release to clamp the tissue oxygenation within a narrow band, which: (1) allows higher frequency measurements of $m{\dot{V}}_{O_{2}}$ during the early phase of recovery; and (2) removes the influence of wide variations in tissue oxygenation. Both benefits are expected to enhance reliability of *k*. Using this method, the occlusion–release duty cycle (analogous to $m{\dot{V}}_{O_{2}}$ sampling frequency) is inherently dependent on the instantaneous rate of $m{\dot{V}}_{O_{2}}$, automatically optimizing the sampling frequency dependent in the underlying *k*. The greater temporal resolution might also be particularly beneficial when the NIRS signal is noisy or attenuated by greater adipose tissue thickness (i.e., an initial arterial occlusion slope of <1.5 % s^−1^). We believe that this is an elegant approach, both to meet the need for a high sampling frequency in the fastest part of the $m{\dot{V}}_{O_{2}}$ recovery kinetics and to prevent wide variations in oxygenation status.

In summary, we encourage the adoption of saturation‐clamp protocols that lessen the potential influence of varying tissue oxygenation on $m{\dot{V}}_{O_{2}}$ recovery kinetics and allow a greater frequency $m{\dot{V}}_{O_{2}}$ measurements that better capture the early phase of the kinetics. Although conventional time‐based protocols might provide similar values of *k* in optimal experimental conditions, saturation‐clamp offers greater experimental control over O_2_ availability and might therefore improve the robustness of the measurement, particularly in technically challenging or physiologically compromised populations. Adoption of such principles should improve the reliability and validity of the NIRS‐based muscle oxidative capacity assessment across studies.

## AUTHOR CONTRIBUTIONS

Alessio Marciano conceived and drafted the manuscript. Alessio Marciano and Harry B. Rossiter edited, revised and approved the final version of this manuscript. All authors agree to be accountable for all aspects of the work in ensuring that questions related to the accuracy or integrity of any part of the work are appropriately investigated and resolved. All persons designated as authors qualify for authorship, and all those who qualify for authorship are listed.

## CONFLICT OF INTEREST

Harry B. Rossiter reports consulting fees from the NIH RECOVER‐ENERGIZE working group (1OT2HL156812) and is involved in contracted clinical research with Biocient, Intervene Immune, Mezzion, Regeneron, Respira and Roche. He is a visiting Professor at the University of Leeds, UK and the University of Pavia, Italy. He is a member of the Scientific Advisory Board for MGC Diagnostics. He reports US/EU patent applications filed by The Lundquist Institute, titled ‘Testing System to Diagnose Neuromuscular Deconditioning and Pathologic Conditions’. He receives royalties as a contributor to the textbook *Wasserman and Whipp's Principles of Exercise Testing and Interpretation*.

## FUNDING INFORMATION

None.

## GENERATIVE AI STATEMENT

Not used.
